
JOURNAL INFORMATION
==============================
NLM Title Abbreviation: Chic Med J
NLM Journal ID: 0027576

The Chicago Medical Journal

Publisher: Biomedical Journal Digitization Project

ARTICLE INFORMATION
==============================
PMCID: PMC9721667
PMID: 37411137
Article version: 1
Subjects: Editorial

Rush Medical College

Print publication date: 1858 Aug
Volume: 15
Issue: 8
First page: 414
Last page: 414
License: This work is free of known copyright restrictions. For more information, please see PMC Back Issue Digitization.
License URL: https://creativecommons.org/publicdomain/mark/1.0/

==============================

APOLOGY AND BILLS.—The undersigned has delayed the issue of the present number, to carry out a determination to personally examine every man's account, and enclose the same in the Journal. It has cost much more time than we expected. The bills are made as they stand on our books. If any subscriber receives one he thinks erroneous, let him immediately inform us and we will be happy to make any corrections required. Let none infer from the delay of this number that we contemplate suspending the Journal. We shall *suspend* it to *those only* who fail to *pay* for it, as stated in the July number. All who *pay* will receive the Journal more promptly in future than any time heretofore.

N. S. DAVIS, *Proprietor*.

RUSH MEDICAL COLLEGE.—Clinical instruction and dissections will commence in connection with this institution on the first Tuesday in October. The Hospital is well filled with patients, and quite a class already in attendance three times a-week. The material for dissection will be abundant. A course of preliminary lectures will also be given during the month of October. The prospects for a good class, and the preparations for their instruction were never better.

### STARLING MEDICAL COLLEGE,

COLUMBUS, OHIO.

### SESSION OF 1858-59.

THE REGULAR SESSION OF STARLING MEDICAL COLLEGE WILL begin on Wednesday, the 20th day of October, 1858, and continue till the first of March.

FACULTY.

S. M. SMITH, M.D., *Professor of Theory and Practice.*
FRANCIS CARTER, M.D., *Professor of Obstetrics, and Diseases of Women and Children.*
J. W. HAMILTON, M.D., *Professor of Surgery.*
JOHN DAWSON, M.D., *Professor of General and Special Anatomy and Physiology, and Dean.*
S. LOVING, M.D., *Prof. of Materia Medica, Therapeutics and Medical Jurisprudence.*
THEO. G. WORMLEY, M.D., *Professor of Chemistry.*
R. N. BARR, M.D., *Demonstrator of Anatomy.*

*FEES.*

| | |
|---|---|
| Tickets of all the Professors, - - - - - | $60 00 |
| Matriculation Ticket, paid but once, - - - | 5 00 |
| Graduation Fee, - - - - - - - - | 20 00 |
| Ticket for the privilege of the Dissecting Room, including the services of the Demonstrator, - - - - | 8 00 |

Subjects for dissection in the building, furnished at a moderate expense, on application to the Demonstrator of Anatomy, *and in no other way*.

Inquiries and requests being sometimes made for indulgence in time, we propose to allow, in such cases, that a judgment note for $65, with interest and approved security payable in one year, may be taken. But our *rule* is payment within the first three weeks of the session.

There are two extensive Bookstores in Columbus, at which Medical works in great variety are sold at very low rates. Surgical, Obstetrical and Dissecting instruments are readily obtained.

☞ All letters of inquiry will receive prompt attention, if addressed to any member of the Faculty, or to **JOHN DAWSON, Dean.**
